# Phactr3/Scapinin, a Member of Protein Phosphatase 1 and Actin Regulator (Phactr) Family, Interacts with the Plasma Membrane via Basic and Hydrophobic Residues in the N-Terminus

**DOI:** 10.1371/journal.pone.0113289

**Published:** 2014-11-18

**Authors:** Akihiro Itoh, Atsushi Uchiyama, Shunichiro Taniguchi, Junji Sagara

**Affiliations:** 1 Department of Biomedical Laboratory Sciences, Health Sciences, Shinshu University, Matsumoto, Japan; 2 Department of Molecular Oncology, Medical Sciences, Shinshu University Graduate School of Medicine, Matsumoto, Japan; Russian Academy of Sciences, Institute for Biological Instrumentation, Russian Federation

## Abstract

Proteins that belong to the protein phosphatase 1 and actin regulator (phactr) family are involved in cell motility and morphogenesis. However, the mechanisms that regulate the actin cytoskeleton are poorly understood. We have previously shown that phactr3, also known as scapinin, localizes to the plasma membrane, including lamellipodia and membrane ruffles. In the present study, experiments using deletion and point mutants showed that the basic and hydrophobic residues in the N-terminus play crucial roles in the localization to the plasma membrane. A BH analysis (http://helixweb.nih.gov/bhsearch) is a program developed to identify membrane-binding domains that comprise basic and hydrophobic residues in membrane proteins. We applied this program to phactr3. The results of the BH plot analysis agreed with the experimentally determined region that is responsible for the localization of phactr3 to the plasma membrane. *In vitro* experiments showed that the N-terminal itself binds to liposomes and acidic phospholipids. In addition, we showed that the interaction with the plasma membrane via the N-terminal membrane-binding sequence is required for phactr3-induced morphological changes in Cos7 cells. The membrane-binding sequence in the N-terminus is highly conserved in all members of the phactr family. Our findings may provide a molecular basis for understanding the mechanisms that allow phactr proteins to regulate cell morphogenesis.

## Introduction

Protein phosphatase 1 (PP1) and actin regulatory (Phactr) proteins are a family that comprises four members in humans and other vertebrates; phactr1–4 [1]. The phactr gene is present in worms and insects but not in protozoa. Accumulating evidence indicates the involvement of phactr proteins in human diseases such as myocardial infarction, Parkinson’s disease, and cancers [2]–[4].

Each phactr protein contains four G-actin-binding RPEL motifs, including an N-terminal motif and a C-terminal triple RPEL repeat. The C-terminal triple RPEL repeat is adjacent to the PP1-binding domain. RPEL motifs are also found in the regulatory domains of myocardin-related transcription factor (MRTF) transcriptional coactivators where they control subcellular localization and activity by sensing signal-induced changes in the G-actin concentration [6], [7]. Subcellular localization of phactr1 that was similar to that of MRTF is controlled by RPEL motifs. Phactr1 exhibits nuclear accumulation in response to serum-induced G-actin depletion [8]. However, there is no evidence for the serum-induced nuclear accumulation of phactr proteins other than phactr1 [8], [9].

Phactr proteins are involved in cell migration both *in vitro* and *in vivo*, and it is believed that phactr is a novel protein family that regulates cytoskeleton dynamics [9]–[14]. However, the mechanisms that regulate actin cytoskeleton dynamics are poorly understood. It has been reported that G-actin and PP1 competitively bind to the C-terminal region and the formation of the phactr–PP1 complex is inhibited by an increase in the cytoplasmic G-actin concentration, which is induced by extracellular signals such as serum. The current hypothesis suggests that the phactr–PP1 complex is controlled by the changes in the cytoplasmic G-actin concentration, which regulate the actin cytoskeleton dynamics by modulating the phosphorylation status of actin regulatory protein(s) [8], [15]. This suggests that phactr proteins regulate both the PP1 activity and subcellular localization by sensing the cytoplasmic actin concentration through RPEL motifs.

The catalytic subunit of PP1 interacts with noncatalytic subunits that determine the activity, substrate specificity, and subcellular localization of the phosphatase [16]. PP1 can dephosphorylate cofilin and myosin [17], [18]. The actin filament-severing activity of cofilin, which stimulates the treadmill-like movement of the actin cytoskeleton in the lamellipodia and filopodia, is controlled by its phosphorylation status, and the force-generating activity of myosin is controlled by the phosphorylation status of myosin itself. In this context, several studies have shown that the phactr–PP1complex modulates the phosphorylation status of cofilin or myosin, and therefore regulating actin cytoskeleton dynamics [8], [12], [15].

Phactr3 was originally named as the nuclear scaffold-associated PP1-inhibiting protein (scapinin), which is found in the nuclear insoluble fraction of the leukemia cell line HL-60 [19]. However, phactr3 is distributed to the plasma membrane in adherent cells, and it enhances cell migration [9]. In the present study, we explored the domains that direct the membrane localization of phactr3 on the basis of deletion and point mutation experiments. We identified a membrane-targeting domain that comprises basic and hydrophobic residues in the N-terminus. This indicates that phactr3 is a membrane-associated PP1 and an actin regulator. Our findings provide a molecular basis for understanding the mechanisms that allow phactr3 to regulate membrane-cytoskeleton dynamics. The amino acid sequences of the N-terminal membrane-targeting domain are highly conserved in the phactr protein family.

## Materials and Methods

### Ethics

This study was carried out in strict accordance with the recommendations in the Guide for the Care and Use of Laboratory Aimals of Shinshu University. The protocol was approved by the Shinshu University Ethics Committee for animal care, handing, termination (Permit Number: 230051). All surgery was performed under Ketamine/xylazine anesthesia, and all efforts were made to minimize suffering.

### GFP-Phactr3 mutants

**Figure 1:**
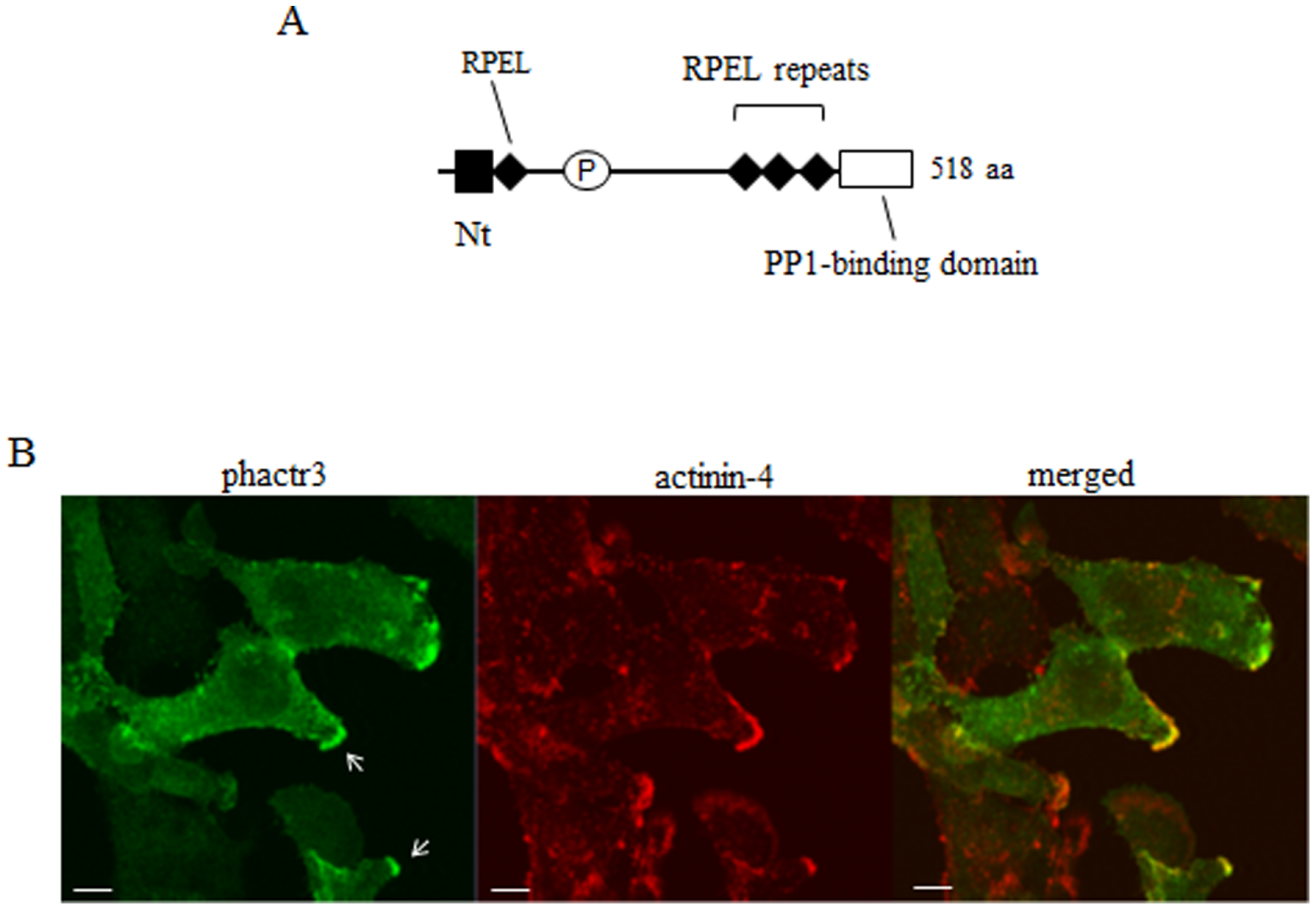
The distribution patterns of phactr3 in cells. (A) Primary structure of phactr3 (NP_001186435, 518 aa). RPEL, G-actin binding motif, and PP1-binding domain (PP1) are indicated. P, a region with a proline-rich sequence. The N-terminal region (Nt), RPEL motifs, and PP1-binding domain are highly conserved in the phactr protein family. (B) Localization of phactr3 in HeLa cells. We have previously established a HeLa cell line where phactr3 expression was induced by tetracycline [5]. The HeLa cells were cultured with tetracycline (0.5 µg/ml) for 24 h and immunostained with anti-phactr3 monoclonal antibody. The distribution patterns of phactr3 were compared with that of α-actinin 4, which is known to localize to the lamellipodia and membrane ruffles [20]. Bars, 20 µm.

The full-length phactr3 cDNA (NP_001186435, 518 aa) was obtained by RT-PCR using an RT primer 5′-AATCTCTATGGCCTGTGGAA-3′, a reverse primer 5′-TCTCTATGGCCTGTGGAATCT-3′, a forward primer 5′-CTGGATGAGATGGACCAAACG-3′, a template poly A^+^ RNA of HL-60 cells, and a high fidelity RNA PCR kit (Takara, Japan). It was subcloned into the *Sma*I site in pKF18k (Takara, Japan). pEGFP-c2-phactr3 was produced by inserting the *Bam*HI/*Eco*RI fragment of pKF18k-phactr3 into *Bgl*II/*Eco*RI sites of pEGFP-c2 (Clontech) [10]. A mutant with a deleted N-terminal region (ΔNt) (Fig. 1C) was generated by PCR-based mutagenesis using a reverse primer 5′-CATCTCATCCAGGGGGATCT-3′ and a forward primer 5′-GCGCTGGAGAAGAAGATGGC-3′. Expand High-Fidelity DNA polymerase (Roche Molecular Biochemicals) was used for the PCR analysis. Deletion mutants of Nt, PP1-binding domain (ΔPP1), ΔRx3/ΔPP1, and NtR (Fig. 1C) were generated by introducing stop codon sequences to terminate protein synthesis at targeted positions using the QuikChange XL site-directed mutagenesis kit (Agilent Technologies). Primers were designed according to the cDNA sequence data of phactr3 (AB098521). The mutations in pEGFP-c2-phactr3 were confirmed by sequencing. Alanine substitution mutants of N1–12 were also generated using the QuikChange XL site-directed mutagenesis kit.

### Cell Culture and Transfection

Cos7 cells (ATCC CRL-1651) were cultured in Dulbecco’s modified minimal essential medium (DMEM) containing 8% fetal bovine serum, 100 units/ml penicillin, and 100 µg/ml streptomycin with 5% CO_2_ and at 37°C. We used FuGENE 6 (Roche Molecular Biochemicals) as the transfection reagent to introduce the plasmid DNA into Cos7 cells. One day before transfection, 0.45 ml of cell suspensions (3×10^5^ cells/ml) were mounted on a silicone-coated two-well glass slide (Matsunami, Japan). Subsequently, 0.5 µg of plasmid DNA (pEGFP-phactr3), its mutants, and 2 µl of FuGENE 6 were mixed in 50 µl of DMEM (antibiotic free) and then allowed to stand for 15 min at room temperature. Twenty microliters of the FuGENE6/DNA mixture was added to each well before cell culture. At 24–48 h post-transfection, 45 µl of formaldehyde (36%–38%) was added to the cell culture to fix the cell morphology. In some cases, the nuclei were stained with Heochst 33342 (1 µg/ml). Fluorescent cells were observed using a BX60-34-FLB-1 fluorescence microscope (Olympus) or an LSM 5 EXCITER confocal laser scanning microscope (Carl Zeiss MicroImaging Inc.). The subcellular localizations of GFP-phactr3 and its mutants were assessed on the basis of three independent experiments.

### Expression of phactr3 in HeLa cells and immunostaining

We established a HeLa cell line using a Tet-ON vector (Invitrogen Life Technology) [9]. Phactr3 expression was induced by the addition of tetracycline at a final concentration of 0.5 µg/ml. After 24 h, the cells were fixed with formaldehyde (3.7%) and immunostained with anti-phactr3 monoclonal antibody [5], followed by FITC-conjugated anti-mouse antibody. Actinin-4 rabbit antibody (rabbit) was generously provided by Dr Yamada T. (National Cancer Center Research Institute, Japan) [20].

### GST-Nt fusion protein

Nt was generated as a fusion protein with glutathione transferase (GST) in *Escherichia coli.* The Nt cDNA was amplified by PCR using a forward primer (5′-CCCGAATTCGATGAGATGGACCAAACGCCCCCG-3′) and a reverse primer (5′-CCCCTCGAGCTACGTTGTCTGCTTCAGTTTTTCGT-3′). The forward primer included an *EcoRI* cleavage site (underlined above), whereas the reverse primer included an *Xho*I cleavage site (CTCGAG) and a stop codon (underlined above). The PCR product was double-digested with *Eco*RI and *Xho*I and integrated between the *Eco*RI and *Xho*I sites of pGEX 4T-1. The BL21 strain of *E. coli* was used to reduce the proteolytic products of a GST fusion protein. GST-Nt was generated by induction with 0.5 mM isopropyl β-D-1-thiogalactopyranoside for 4 h at 25°C and purified with glutathione-Sepharose 4B beads (GE Healthcare).

### Liposome co-sedimentation assay

All surgery was performed under Ketamine/xylazine anesthesia, and all efforts were made to minimize suffering. Rat brains were homogenized in a methanol/chloroform mixture that included acetic acid, and the total lipid fraction was extracted and dried with nitrogen gas [21]. The dried lipids were hydrated in a binding buffer (25 mM HEPES-NaOH, pH 7.4, 100 mM NaCl, and 0.5 mM EDTA) at a final concentration of 2 mg/ml by shaking for 60 min at 37°C. The hydrated lipid sample was then sonicated in a bath sonicator to generate liposomes. A liposome co-sedimentation assay was performed [22]. The GST-Nt and GST solutions were centrifuged for 90 min at 100,000×*g* at 4°C to remove any aggregates. The protein concentrations were measured using a BCA protein assay kit (Pierce) and diluted to 100 µg/ml. Subsequently, 250 µl of the liposome and protein solutions, respectively, were mixed and incubated for 20 min at room temperature. The protein/liposome mixture was centrifuged for 60 min at 100,000×*g* at room temperature to yield the supernatant (sup) and precipitate (ppt). ppt was resuspended in 500 µl of binding buffer. Aliquots of sup and ppt were subjected to SDS-PAGE and stained with Coomassie brilliant blue.

### Lipid-binding assay with Membrane Lipid Strips

PIP strips and Membrane Lipid Strips (Echelon Biosciences Inc.) were used in this study. The membrane strips were prespotted with various lipids, and lipid-binding assays were performed [23]. 100 moles of each of the following lipids were spotted: lysophosphatidic acid (LPA), lysophosphocholine (LPC), phosphatidylinositol (PI), phosphatidylinositol-(3)-phosphate (PI(3)P), phosphatidylinositol-(4)-phosphate (PI(4)P), phosphatidylinositol-(5)-phosphate (PI(5)P), phosphatidylethanolamine (PE), phosphatidylcholine (PC), sphingosine-1-phosphate (SIP), phosphatidylinositol-(3,4)-bisphosphate (PI(3,4)P_2_), phosphatidylinositol-(3,5)-bisphosphate (PI(3,5)P_2_), phosphatidylinositol-(4,5)-bisphosphate (PI(4,5)P_2_), phosphatidylinositol-(3,4,5)-trisphosphate (PI(3,4,5)P_3_), phosphatidic acid (PA), and phosphatidylserine (PS). The strips were blocked with 3% bovine serum albumin in PBS-T [PBS (−) containing 0.02% Tween 20] and then incubated with GST-Nt or GST at a protein concentration of 0.5 µg/ml. GST-Nt and GST were detected using rabbit anti-GST antibody (MBL, Japan) and horseradish peroxidase-conjugated anti-rabbit immunoglobulin (the second antibody). Finally, the strips were immersed in a solution of Immobilon Western Chemiluminescent HRP Substrate (Merck Millipore) and exposed to X-ray films.

## Results

### Phactr3 localizes to the plasma membrane through its N-terminal region (Nt)

**Figure 2:**
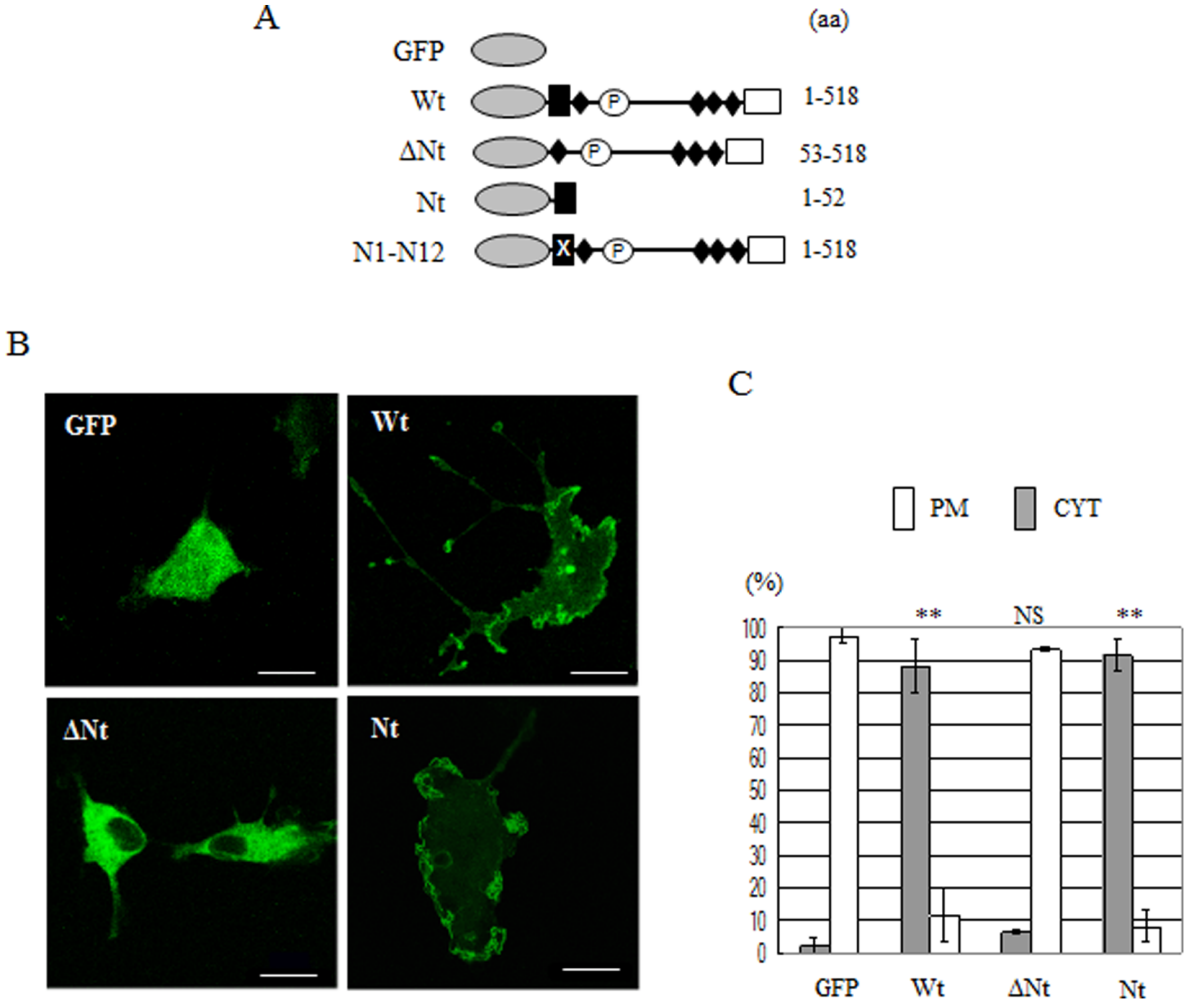
The N-terminal region (Nt) is responsible for the distribution of phactr3 in the plasma membrane. (A) Deletion mutants of GFP-phactr3 used in this study. (B) GFP and GFP-phactr3 constructs were expressed in Cos7 cells and their subcellular distributions were observed by confocal laser scanning microscopy. Bars, 20 µm. (C) The numbers of cells with predominantly plasma membrane (PM) distributions and cytoplasm (CYT) distributions were counted by fluorescence microscopy. The mean and standard error (SE) were calculated on the basis of three independent experiments (shown in the figure as mean ± SE). The asterisks in the histograms indicate significant differences between Wt and each mutant (Student’s t-test; **P<0.005). NS, not significant.

The entire sequence of Nt (1–52 aa), the functional roles of which are unknown, is encoded by exon2 of the *phactr3* gene (Fig. 1A). In HeLa cells, phactr3 was distributed throughout the cells, but it was frequently localized to the plasma membrane including the lamellipodia and membrane ruffles (Fig. 1B). Figure 1B compares its distribution pattern with that of α-actinin 4, which localizes to the lamellipodia and membrane ruffles [20]. In the present study, we introduced deletions and point mutations into GFP-phactr3 (Fig. 1) to determine the domain responsible for localization to the plasma membrane. GFP-phactr3 constructs were expressed in Cos7 cells and their distribution patterns were observed by fluorescent microscopy and confocal laser scanning microscopy. GFP-Phactr3-Wt was distributed in the plasma membrane including the lamellipodia and membrane ruffles [9]. The distribution pattern of GFP-phactr3-Wt in Cos7 cells was essentially the same as that of phactr3 (no tag) in HeLa cells (Fig. 1B and 2B).

ΔNt greatly impaired localization to the plasma membrane. However, Nt could be localized to the plasma membrane by itself (Fig. 2). These results demonstrate that the localization of phactr3 to the plasma membrane requires Nt.

In addition to the lamellipodia and membrane ruffles, GFP-phactr3-Wt was often distributed in long/slender cytoplasmic extensions in Cos7 cells where it was concentrated at the tips of these extensions (Fig. 2B). We also analyzed these cytoplasmic extensions using mutants (see below).

### N-terminal basic and hydrophobic residues are crucial for membrane targeting

**Figure 3:**
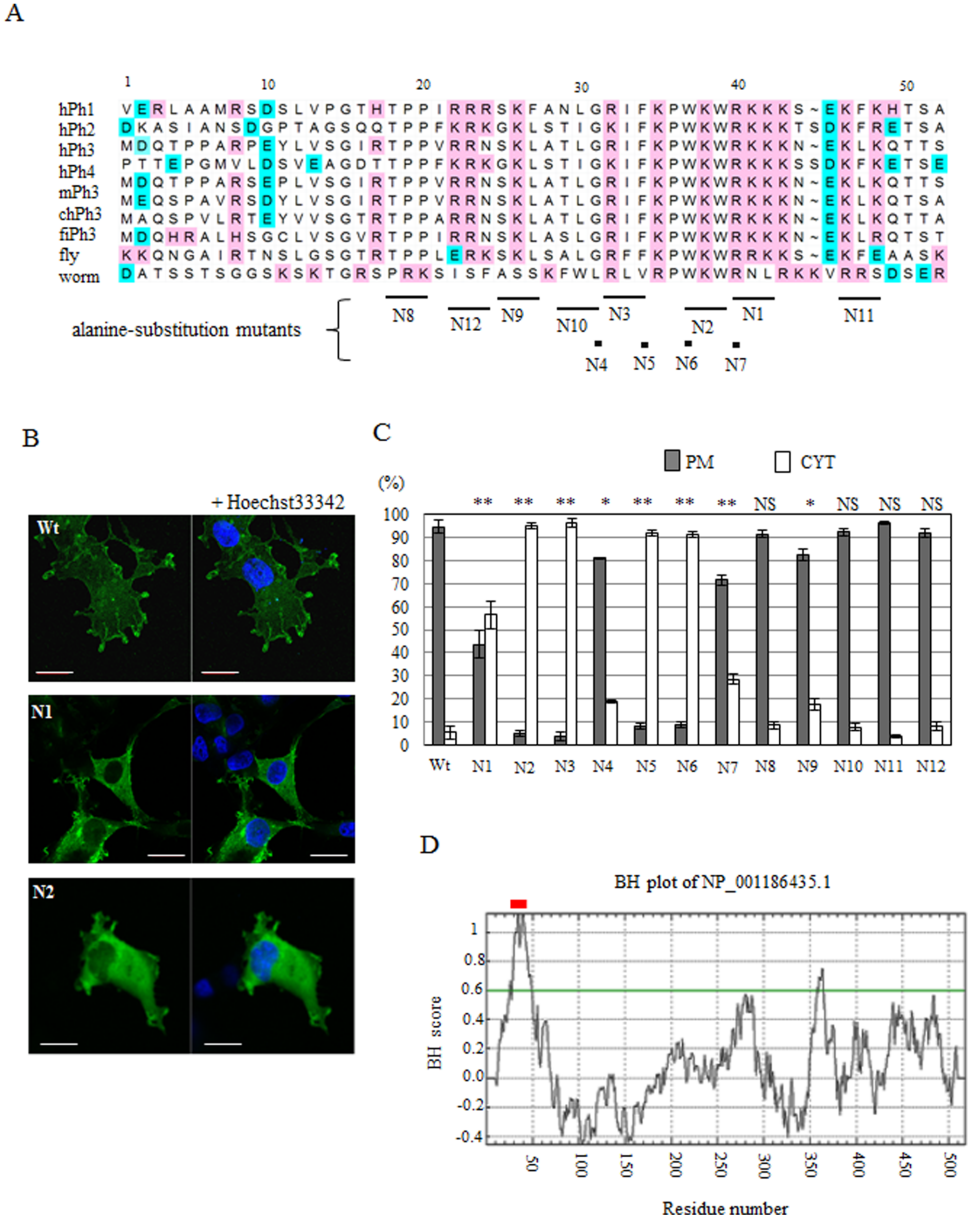
Basic and hydrophobic residues in the Nt are responsible for the distribution in the plasma membrane. (A) Alignment of the Nt amino acid (aa) sequences of phactr. Human phactr 1–4 (hPh1–4), mouse phactr3 (mouse), chicken phactr3 (bird), zebra fish phactr3 (fish), *Drosophila melanogaster* phactr (fly), and *Caenorhabditis elegans* phactr (worm) were aligned. N1–12 indicate substituted with alanine residues. (B) The distribution patterns of GFP-phactr3 Wt, N1, and N2. Cos7 cells expressing each construct were photographed using confocal laser microscopy at 24 h after transfection. The nucleus was stained with Hoechst 33342. Bars, 20 µm. (C) Amino acid residues in the indicated Nt (A) were substituted with alanine residues and expressed in Cos7 cells. The numbers of cells with predominantly plasma membrane (PM) distributions and cytoplasm (CYT) distributions were counted by fluorescence microscopy [(denoted as mean ± standard error (SE)]. The asterisks in the histograms indicate significant differences between Wt and each mutant (Student’s t-test; *P<0.05, **P<0.005). NS, not significant. (D) The BH plot analysis of the membrane interaction domains [24] detected a potent membrane interaction domain in Nt. Red bar indicates the experimentally deduced region responsible for the distribution in the plasma membrane.

To determine the N-terminal amino acid (aa) sequence required for the interaction with the plasma membrane, we substituted the aa residues in Nt with alanine (N1–12; Fig. 3A) and observed subcellular distributions. For example, the N1 (RKK>AAA) and N2 (WKW>AAA) mutants were predominantly distributed in the cytoplasm (Fig. 3B). We counted the number of cells that predominantly exhibited plasma membrane distributions or cytoplasm distributions using a fluorescent microscopy. In the triplet mutants N1, N2, N3, and N9, and the point mutants N4, N5, N6, and N7, the number of cells that localized to the plasma membrane was greatly or moderately reduced (Fig. 3C). These mutation experiments demonstrated that aa sequence of GRIFKPWKWRKK (31–42 aa) in Nt is crucial for localization to the plasma membrane. In addition, the peptide of SKL (25–27 aa) may be involved in membrane targeting.

A BH plot analysis (http://helixweb.nih.gov/bhsearch) is a program developed to identify or predict unstructured membrane-binding domains that comprise basic and hydrophobic residues in membrane proteins [24]. Therefore, we applied this program to phactr3 (HP_001186435, 518 aa). The BH plot analysis detected a peak score in Nt (Fig. 3D). The region with a BH score of >0.8 ranged from 29 to 45 aa, and the region with a BH score of >0.6 ranged from 25 to 49 aa. The results of the BH plot analysis agreed with the experimentally determined region that is responsible for the localization of phactr3 to the plasma membrane. Furthermore, the BH plot analysis indicated that a wide range of N-terminal polypeptides, including GRIFKPWKWRKK (31–42 aa), may be involved in the membrane targeting of phactr3. However, we could not exclude the possibility of contributions by other regions. A weak membrane-targeting signal is present within the C-terminal RPEL repeats (Fig. 3D).

### Interaction between Nt and lipid layers

The following two possible molecular mechanisms may allow Nt to interact with the plasma membrane: direct or indirect binding (binding via other membrane proteins) with lipid bilayers. The BH search results indicated that Nt interacts directly with the lipid bilayers [24].

**Figure 4:**
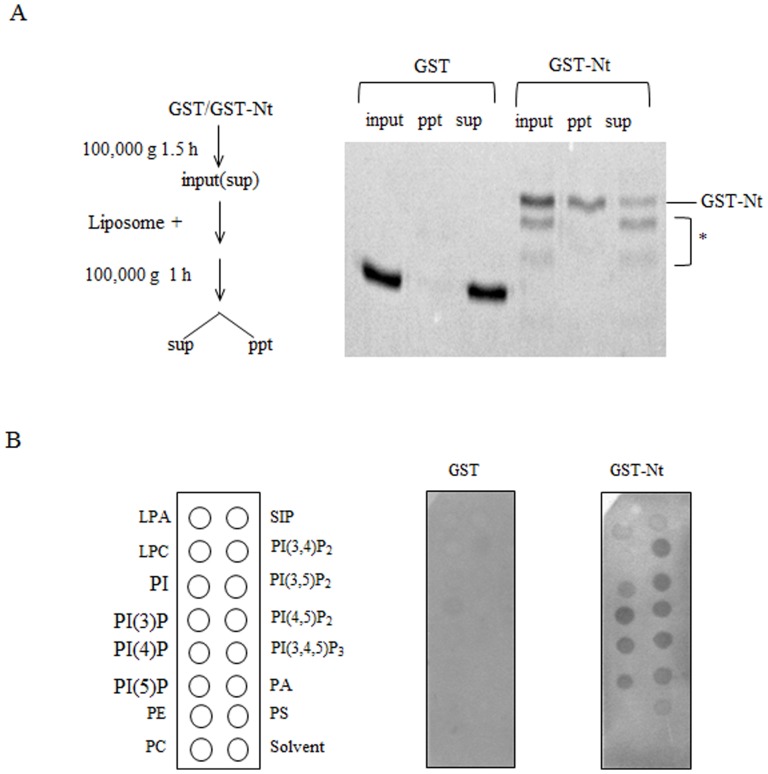
The interaction between the Nt and lipid bilayers. (A) Liposome co-sedimentation assay. Nt was produced as a fusion protein with glutathione transferase (GST-Nt) in the BL21 strain of *Escherichia coli* and purified with glutathione-Sepharose 4B beads. GST was used as a control. Diagram showing the liposome sedimentation assay. GST and and GST-Nt were centrifuged before use to sediment insoluble proteins and mixed with liposomes (input). After centrifugation, the liposome-bound (ppt) and liposome-unbound (sup) fractions were recovered. Aliquots of each fraction were separated on an SDS-PAGE and stained with Coomassie brilliant blue. (B) Lipid binding assay using the spot array method. Lipid-spotted membrane strips were incubated with GST or GST-Nt and the lipid-bound proteins were detected using anti-GST antibody. LPA, lysophosphatidic acid; PI, phosphatidylinositol; PI(3)P, phosphatidylinositol-(3)-phosphate; PI(4)P, phosphatidylinositol-(4)-phosphate; PI(5), phosphatidylinositol-(5)-phosphate; PE, phosphatidylethanolamine, PC, phosphatidylcholine; SIP, sphingosine-1-phosphate; PI(3,4)P_2_; phosphatidylinositol-(3,4)-bisphosphate; PI(3,5)P_2_, phosphatidylinositol-(3,5)-bisphosphate; PI(3,4,5)P_3_, phosphatidylinositol-(3,4,5)-trisphosphate; PA, phosphatidic acid; PS, phosphatidylserine.

We examined whether Nt directly binds to lipid bilayers using a liposome co-sedimentation assay. First, the insoluble proteins were precipitated by centrifugation. Subsequently, GST-Nt (1–52 aa) or GST (control) were mixed with liposomes and separated into liposome (ppt) and soluble (sup) fractions by centrifugation (Fig. 4A). GST-Nt sedimented with the liposomes, whereas GST did not. It was notable that the cleaved products did not sediment with the liposomes. Although we used the BL21 strain of *E. coli* to express GST-Nt and to reduce proteolytic products, the GST-Nt samples always included cleaved products (star-shaped products). Therefore, it appears that Nt is highly sensitive to cleavage by the endogenous bacterial protease.

Subsequently, we examined the species of lipids that interact with Nt using lipid spot array filters (Fig. 4B). This assay showed that Nt predominantly bound to acidic phospholipids but not with neutral phospholipids such as phosphatidylcholine and phosphatidylethanolamine. Nt did not bind to cholesterol and phosphatidylglycerol (data not shown).

### Stimulation of the formation of long/slender cytoplasmic extensions in Cos7 cells

**Figure 5:**
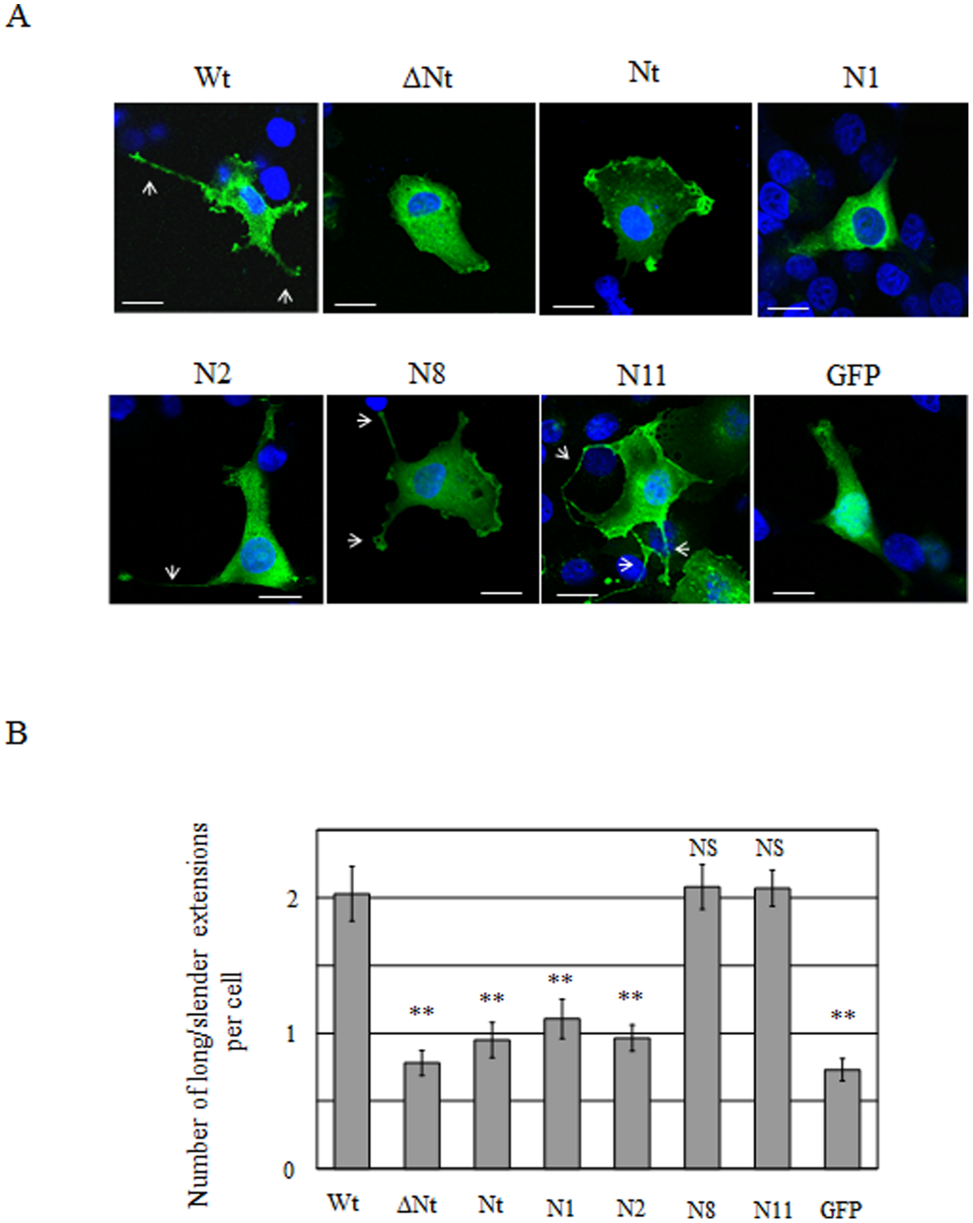
Long/slender cytoplasmic extensions induced by the expression of GFP-phactr3 and its mutants in Cos 7 cells. (A) Distribution patterns of GFP-phactr3 Wt and mutants in Cos7 cells. Cos7 cells expressing Wt or each mutant were imaged using confocal laser microscopy at 24 h after transfection. The nucleus was stained with Hoechst 33342. The arrows show long/slender cytoplasmic extensions. (B) The number of long/slender extensions in Cos7 cells expressing each mutant. Each plasmid was transfected into Cos7 cells and the number of long/slender extensions per cell was counted by fluorescent microscopy at 24 h after transfection. Mean ± standard error (SE). The asterisks in the histograms indicate significant differences between Wt and each mutant (Student’s t-test; **P<0.005). NS, not significant.

GFP-phactr3-Wt was often distributed in long/slender cytoplasmic extensions in Cos7 cells, where it was frequently concentrated at the tips of these extensions (Fig. 2B and 5A). We have previously shown that phactr3-expressing Hela cells frequently exhibited elongated shapes that were rarely seen in the parental Hela cells [9]. However, the long/slender extensions were not seen in HeLa cells that expressed phactr3 (no tag) and GFP-phactr3-Wt [9], [19]; therefore, indicating that the formation of these structures is cell type-dependent. Long/slender cytoplasmic extensions were observed in Cos7 cells that expressed GFP, although the number of cytoplasmic extensions was less than that with GFP-phactr3-Wt. We compared the number of cytoplasmic extensions in Cos7 cells that expressed wild-type or mutant phactr3 at 24 h after transfection (Fig. 5B). The number of cytoplasmic extensions was reduced in Cos7 cells that expressed N1 and N2 (mutants with incomplete membrane-targeting sequences), but it was not reduced in Cos7 cells that expressed N8 and N11 (mutants with the complete membrane-targeting sequence). In addition, the N10 and N12 mutants with complete membrane-targeting sequences stimulated the formation of long/slender cytoplasmic extensions, whereas the N3, N5, N6, and N7 mutants with incomplete membrane-targeting sequences failed to stimulate the formation of long/slender cytoplasmic extensions (data not shown). Nuclear morphologies that are characteristic to apoptosis or necrosis were not seen in Cos7 cells that expressed wild-type or mutants of phactr3. These results indicate that the integrity of the membrane-targeting sequence in the N-terminus is required to stimulate the formation of long/slender cytoplasmic extensions in Cos7 cells. It should be noted that GFP-Nt did not stimulate the formation of these cytoplasmic extensions; therefore, implying that Nt is sufficient for membrane targeting whereas it is insufficient for stimulating the formation of long/slender cytoplasmic extensions in Cos7 cells.

## Discussion

The phactr protein family is considered to be involved with cell migration and morphogenesis by modulating the actin cytoskeleton, but their regulatory mechanisms are poorly understood [8]–[10], [12]. Our experiments with deletion mutants demonstrated that Nt is required for the localization of phactr3 to the plasma membrane (Fig. 2).

Phactr3 lacks fatty acid-attachment sites (e.g., palmitoylation, prenylation, and myristorylation sites) and lipid-binding domains with defined ternary structures (e.g., a PH domain). The mutation experiments showed that the N-terminal peptide (31–42 aa), which comprises basic and hydrophobic residues, is critical for localization to the plasma membrane (Fig. 3C). The BH plot analysis, a program that identifies membrane-targeting domains containing unstructured clusters of basic and hydrophobic residues in membrane proteins, supported this result (Fig. 3D). The BH plot analysis also indicated that the N-terminal sequence can directly interact with lipid bilayers without any requirement for other membrane proteins. We also performed an *in vitro* analysis and showed that Nt interacts with the liposome without the involvement of other membrane proteins (Fig. 4A); therefore, demonstrating the direct interaction between phactr3 and lipid bilayers.

The region with a BH score of >0.8 ranged from 29 to 45 aa, and the region with a BH score of >0.6 ranged from 25 to 49 aa. This indicates that although the 31–42 aa sequence in Nt is the most critical for interactions, other aa residues adjacent to the critical sequence may be involved in membrane interactions. Analyses using synthetic peptides are necessary to determine the precise ranges of the membrane-interacting domains.

Point mutations of phenylalanine (N5) or tryptophan (N6) in the membrane interaction sequence resulted in impaired localization to the plasma membrane. This indicates that these hydrophobic residues play critical roles in the interactions with the plasma membrane possibly by inserting their aromatic head groups into lipid bilayers. Analyses using lipid blot array filters demonstrated that Nt of phactr3 binds to a broad range of acidic phospholipids (Fig. 4B). It is well known that the clusters of basic residues in membrane proteins are involved in electrostatic interactions with the negative surface charge on the inner leaflet of the plasma membrane [25]–[27]. The aa sequence of GRIFKPWKWRKK (31–42 aa) was shown to be critical for localization to the plasma membrane and it includes six basic residues (Fig. 3A). Nt with a BH score of >0.6 included 10 basic residues.

Some actin regulatory proteins, such as N-WASP, WAVE, profilin, and cofilin, selectively bind to specific species of phosphoinositides such as phosphatidylinositol-(4,5)-bisphosphate and phosphatidylinositol-(3,4,5)-trisphosphate [28]–[29]. The selective recognition of phospholipids facilitates the controlled (spatial and temporal) recruitment of these actin regulatory proteins to specific membrane domains. However, Nt of phactr3 nonselectively binds to negatively charged phospholipids. This nonselective recognition mode may allow the constitutive recruitment of phactr3 to the plasma membrane and/or intracellular membrane [27].

The experimentally determined membrane-targeting sequence is highly conserved in phactr proteins from insects to humans and partly conserved in that from worms (Fig. 3A). The BH plot analysis showed that the highly or partly conserved sequence of all members of the phactr family is probably the membrane-binding site. Huet et al., [15] demonstrated that phactr4 localizes to the plasma membrane through its Nt (1–111 aa). They measured the Forster resonance energy transfer between GFP-Phactr4 and mCherry-CAAX (a plasma membrane probe) to assess the interaction between phactr4 and the plasma membrane. Their conclusion appears to be in agreement with our results, although they did not characterize the membrane interaction sequence. Therefore, it is likely that the N-terminal conserved sequence acts as the membrane-targeting sequence in all members of the phactr family.

GFP-phactr3-Wt stimulated the formation of long/slender cytoplasmic extensions in Cos7 cells (Fig. 2B and 5). GFP-phactr3 mutants with incomplete membrane-targeting sequences failed to stimulate the formation of these cytoplasmic extensions, whereas GFP-phactr3 mutants with the complete membrane-targeting sequence resulted in morphological changes that were similar to those with GFP-phactr3-Wt. GFP-Nt did not increase the number of long/slender cytoplasmic extensions (Fig. 5); therefore, implying that Nt is sufficient for membrane targeting but that it is insufficient to stimulate the formation of long/slender cytoplasmic extensions in Cos7 cells (Fig. 5). Therefore, the interactions with PP1 and actin via the C-terminal region and the interaction with the plasma membrane via Nt may be necessary for inducing morphological changes in Cos7 cells.

G-actin and PP1 competitively bind to the C-terminal regions of phactr proteins and the cytoplasmic G-actin concentration is determined by RPEL motifs, which control the formation of the phactr-PP1 complex [8], [15]. Previous studies suggest that the phactr-PP1complex modulates the phosphorylation status of cofilin or myosin; therefore, regulating actin cytoskeleton dynamics [8], [12], [15]. Overall, these results suggest that phactr protein is a membrane-associated PP1 regulator, which is itself regulated by signal-induced changes in the cytoplasmic G-actin levels. phactr1 modulates lamellipodial dynamics in human endothelial cells, which are regulated by dynamic interactions between the plasma membrane and actin cytoskeleton [13]. Therefore, it is likely that the phactr-PP1 complex modulates the phosphorylation status of actin cytoskeleton regulators such as cofilin and myosin, which associate with the plasma membrane.

## References

[1] Allen PB, Greenfield AT, Svenningsson P, Haspeslagh DC, Greengard P (2004) Phactrs 1–4: A family of protein phosphatase 1 and actin regulatory proteins. Proc. Natl. Acad. Sci. U.S.A. 101: 7187–7192 10.1073/pnas.0401673101 PMC406487 15107502

[2] Myocardial infraction Consortium (2009) Genome-wide association of early-onset myocardial infarction with single nucleotide polymorphisms and copy number variants. Nat. Genet. 41: 334–341 10.1038/ng.327 PMC2681011 19198609

[3] Wider C, Lincoln SJ, Heckman MG, Diehl NN, Stone JT, et al (2009) Phactr2 and Parkinson’s disease. Neurosci Lett 453: 9–11 10.1016/j.neulet.2009.02.009 19429005 PMC2684848

[4] Solimini NL, Liang AC, Xu C, Pavlova NN, Xu Q, et al (2013) STOP gene Phactr4 is a tumor suppressor. Proc Natl Acad Sci USA 110: E407–414 10.1073/pnas.1221385110 23319639 PMC3562831

[5] Mouilleron S, Wiezlak M, O’Reilly N, Treisman R, McDonald NQ (2012) Structures of the Phactr1 RPEL domain and RPEL motif complexes with G-actin reveal the molecular basis for actin binding cooperativity. Structure 20: 1960–1970 10.1016/j.str.2012.08.031 23041370

[6] Miralles F, Posern G, Zaromytidou AU, Treisman R (2003) Actin dynamics control SRF activity by regulation of its coactivator MAL. Cell 113: 329–342 10.1016/S0092-8674(03)00278-2 12732141

[7] Vartiainen MK, Guettler S, Larijani B, Treisman R (2007) Nuclear actin regulates dynamic subcellular localization and activity of the SRF cofactor MAL. Science 316: 1749–1752 10.1126/science.1141084 17588931

[8] Wiezlak M, Diring J, Abella J, Mouilleron S, Way M, et al (2012) G-actin regulates the shuttling and PP1 binding of the RPEL protein Phactr1 to control actomyosin assembly. J Cell Sci 125: 5860–5872 10.1242/jcs.112078 22976292

[9] Sagara J, T. Arata T, Taniguchi S (2009) Scapinin, the protein phosphatase 1 binding protein, enhances cell spreading and motility by interacting with the actin cytoskeleton. PLoS One 4: e4247 10.1371/journal.pone.0004247 19158953 PMC2626280

[10] Favot L, Gillingwater M, Scott C, Kemp PR (2005) Overexpression of a family of RPEL proteins modifies cell shape. FEBS Lett 579: 100–104 10.1016/j.febslet.2004.11.054 15620697

[11] Farghaian H, Chen Y, Fu AW, Fu AK, Ip JP, et al (2011) Scapinin-induced inhibition of axon elongation is attenuated by phosphorylation and translocation to the cytoplasm. J Biol Chem 286: 19724–19734 10.1074/jbc.M110.205781 21487013 PMC3103351

[12] Zhang Y, Kim TH, Niswander L (2012) Phactr4 regulates directional migration of enteric neural crest through PP1, integrin signaling, and cofilin activity. Genes Dev 26: 69–81 10.1101/gad.179283.111 22215812 PMC3258968

[13] Allain B, Jarray R, Borriello L, Leforban B, Dufou S, et al (2012) Neuropilin-1 regulates a new VEGF-induced gene, Phactr-1, which controls tubulogenesis and modulates lamellipodial dynamics in human endothelial cells. Cell Signal 24: 214–223 10.1016/j.cellsig.2011.09.003 21939755

[14] Fils-Aimé N, Dai M, Guo J, El-Mousawi M, Kahramangil B, et al (2013) MicroRNA-584 and the protein phosphatase and actin regulator 1 (PHACTR1), a new signaling route through which transforming growth factor-β Mediates the migration and actin dynamics of breast cancer cells. J Biol Chem 288: 11807–11823 10.1074/jbc.M112.430934 23479725 PMC3636869

[15] Huet G, Rajakylä EK, Viita T, Skarp KP, Crivaro M, et al (2013) Actin-regulated feedback loop based on Phactr4, PP1 and cofilin maintains the actin monomer pool. J Cell Sci 126: 497–507 10.1242/jcs.113241 23203801

[16] Bollen M, Stalmans W (1992) The structure, role, and regulation of type 1 protein phosphatases, Crit. Rev. Biochem. Mol Biol 27: 227–281 10.3109/10409239209082564 1350240

[17] Alessi D, MacDougall LK, Sola MM, Ikebe M, Cohen P (1992) The control of protein phosphatase-1 by targetting subunits. The major myosin phosphatase in avian smooth muscle is a novel form of protein phosphatase-1. Eur J Biochem 210: 1023–1035 10.1111/j.1432-1033.1992.tb17508.x 1336455

[18] Ambach A, Saunus J, Konstandin M, Wesselborg S, Meuer SC, et al (2000) The serine phosphatases PP1 and PP2A associate with and activate the actin-binding protein cofilin in human T lymphocytes. Eur J Immunol 30: 3422–3431 doi:;10.1002/1521-4141(2000012)30: 12<3422::AID-IMMU3422>3.0.CO; 2-J 11093160 10.1002/1521-4141(2000012)30:12<3422::AID-IMMU3422>3.0.CO;2-J

[19] Sagara J, Higuchi T, Hattori Y, Moriya M, Sarvotham H, et al (2003) Scapinin, a putative protein phosphatase-1 regulatory subunit associated with the nuclear nonchromatin structure. J Biol Chem 278: 45611–45619 10.1074/jbc.M305227200 12925532

[20] Honda K, Yamada T, Endo R, Ino Y, Gotoh M, et al (1998) Actinin-4, a novel actin-bundling protein associated with cell motility and cancer invasion. J Cell Biol 140: 1383–1393 10.1083/jcb.140.6.1383 9508771 PMC2132673

[21] Bligh EG, Dyer WJ (1959) A rapid method of total lipid extraction and purification. Can J Biochem Physiol 37: 911–917 10.1139/o59-099 13671378

[22] Patki V, Virbasius J, Lane WS, Toh BH, Shpetner HS, et al (1997) Identification of an early endosomal protein regulated by phosphatidylinositol 3-kinase. Proc Natl Acad Sci USA 94: 7326–7330. 9207090 10.1073/pnas.94.14.7326 PMC23820

[23] Kavran JM, Klein DE, Lee A, Falasca M, Isakoff SJ, et al (1998) Specificity and promiscuity in phosphoinositide binding by pleckstrin homology domains. J Biol Chem 273: 30497–30508 10.1074/jbc.273.46.30497 9804818

[24] Brzeska H, Guag J, Remmert K, Chacko S, Korn ED (2010) An experimentally based computer search identifies unstructured membrane-binding sites in proteins: application to class I myosins, PAKS, and CARMIL. J Biol Chem 285: 5738–5747 10.1074/jbc.M109.066910 20018884 PMC2820801

[25] Niggli V (2001) Structural properties of lipid-binding sites in cytoskeletal proteins. Trends Biochem Sci 26: 604–611 10.1016/S0968-0004(01)01927-2 11590013

[26] McLaughlin S, Murray D (2005) Plasma membrane phosphoinositide organization by protein electrostatics. Nature 438: 605–611 10.1038/nature04398 16319880

[27] Moravcevic K, Oxley CL, Lemmon MA (2012) Conditional peripheral membrane proteins: facing up to limited specificity. Structure 20: 15–27 10.1016/j.str.2011.11.012 22193136 PMC3265387

[28] Yin HL, Janmey PA (2003) Phosphoinositide regulation of the actin cytoskeleton. Annu Rev Physiol 65: 761–789 10.1146/annurev.physiol.65.092101.142517 12471164

[29] Takenawa T, Suetsugu S (2007) The WASP-WAVE protein netwaok: connecting the membrane to the cytoskeleton. Nat Rev Mol Cell Biol 8: 37–48 10.1038/nrm2069 17183359

